# IL-10 Plays a Critical Role in Mitigating Acute Anaemia Development During African Trypanosome Infection

**DOI:** 10.3390/pathogens14121276

**Published:** 2025-12-12

**Authors:** Maida Živalj, Anaïs St. Martin, Patrick De Baetselier, Liudmyla Maksymova, Fara Berghmans, Louis Boon, Jo A. Van Ginderachter, Stefan Magez, Carl De Trez, Benoit Stijlemans

**Affiliations:** 1 Laboratory of Cellular and Molecular Immunology, Brussels Center for Immunology, Vrije Universiteit Brussel, 1050 Brussels, Belgium; maida.zivalj@vub.be (M.Ž.); anais.stmartin@hotmail.be (A.S.M.); patrick.de.baetselier@vub.be (P.D.B.); jo.van.ginderachter@vub.be (J.A.V.G.); stefan.magez@vub.be (S.M.); carl.de.trez@gmail.com (C.D.T.); 2 Myeloid Cell Immunology Laboratory, VIB Center for Inflammation Research, 1050 Brussels, Belgium; 3 JJP Biologics, 00-728 Warsaw, Poland; louis.boon@jjp.bio

**Keywords:** IL-10, erythropoiesis, anaemia, IFN-γ, central macrophage

## Abstract

During the first week of *T. b. brucei* infection, pro-inflammatory IFN-γ production drives acute anaemia by promoting red blood cell clearance by activated macrophages in concert with insufficient bone marrow compensation. The latter is followed by a partial recovery phase, which later progresses to chronic anaemia. To compensate for acute anaemia, stress-induced extramedullary erythropoiesis occurs in the spleen. However, the role of IL-10, a key anti-inflammatory cytokine in regulating stress-induced acute anaemia during African trypanosomosis (AT), remains unclear. Using both genetic and pharmacological approaches, we show that IL-10 is essential to limit acute anaemia by dampening inflammation and promoting splenic erythropoiesis, enabling recovery. More specifically, IL-10 blockade impairs erythropoiesis in both bone marrow and spleen, particularly at early erythroid differentiation stages, and associates with reduced central macrophage (CM) numbers in the bone marrow. In contrast, the co-inhibition of IL-10 and IFN-γ reduces inflammation and partially restores splenic CM numbers and erythropoiesis, highlighting IFN-γ’s suppressive role in erythropoiesis. Overall, these findings underscore IL-10’s key role in regulating stress-induced erythropoiesis during AT by modulating erythroid differentiation and CM abundance, thereby limiting immune-mediated acute anaemia. Consequently, timely adjustment of the IL-10/IFN-γ balance may enhance erythropoiesis and offer a potential therapeutic strategy to mitigate anaemia development.

## 1. Introduction

African trypanosomosis (AT), one of the most ‘neglected tropical diseases’, is transmitted by tsetse flies (*Glossina* ssp.). Human African trypanosomosis (HAT), also called the ‘sleeping sickness’, is endemic in sub-Saharan Africa. The causative agents of HAT belong to the *Trypanosoma brucei* (*T. b.*) species, which consists of three subspecies. *T. b. gambiense* (located in Western and Central Africa and causing chronic infection) and *T. b. rhodesiense* (present in Eastern and Southern Africa and causing acute infection) infect humans [1], but can also infect domestic and wild animals that serve as reservoirs [2,3], whereas the third species, *T. b. brucei*, strictly infects animals. When animals get infected, the disease is called Animal African Trypanosomosis (AAT), or ‘Nagana’. AAT causes significant economic losses on the African continent, primarily through reduced livestock productivity and increased costs of control, prevention, and treatment. The disease manifests with a range of pathological features, including anemia, intermittent fever, edema, and weight loss, whereby its severity can vary depending on the Trypanosoma species involved and the animal species affected. One of the most important pathological features associated with AAT is anemia, where trypanotolerance is characterized by animals being able to control anemia development [4,5]. Moreover, the key difference between susceptible and tolerant animals lies in their ability to mount an effective compensatory erythropoiesis. Murine models offer a powerful platform to study experimental trypanosome infections, enabling detailed analysis of parasite–host immune interactions, anemia development [5], and the genetic factors that influence disease susceptibility and progression [6].

It was shown that the early phase of trypanosome infections is characterized by an initial, most prominent, parasitaemia peak, which triggers a strong type 1 immune response. In this context, the IFN-γ production is a driving force for the induction of the inflammatory immune response, resulting in the activation of myeloid cells and the subsequent release of other pro-inflammatory molecules such as TNF [7]. While essential for controlling peak parasitemia, the strong pro-inflammatory immune response also triggers a cascade that leads to acute anaemia, which is also referred to as consumptive anaemia of inflammation and resembles the human hemophagocytic syndrome [5,8]. After this phase, there is a partial recovery phase, followed by a second and more progressive phase of anaemia, which exhibits characteristics of anaemia of inflammation [9]. The underlying mechanisms involved in anaemia progression during *T. brucei* infection are complex and multifactorial, involving an altered inflammatory immune response, an altered iron homeostasis, and an imbalance between erythrophagocytosis and erythropoiesis [10,11].

As mentioned before, IFN-γ is an important cytokine promoting the induction of inflammation and acute anaemia by stimulating the activation of myeloid cells [12]. These activated cells exhibit an enhanced ability to phagocytose damaged red blood cells (RBCs), resulting in the induction of acute anaemia [7]. Due to increased erythrophagocytosis and the bone marrow’s inability to rapidly compensate via erythropoiesis, acute anaemia develops. To counter this, extramedullary erythropoiesis (EME) is initiated in the spleen, leading to splenomegaly [10]. This stress-induced process relies on stress erythroid progenitors (SEPs), derived from short-term reconstituting hematopoietic stem cells that are distinct from steady-state progenitors. Indeed, SEPs respond to signals such as Hedgehog, Bmp4, and Gdf15, pathways that are not involved in normal erythropoiesis [13,14,15,16]. These signals drive the expansion of immature SEPs, which retain stem cell markers and act as a transient amplifying pool. Elevated erythropoietin (Epo) then promotes their differentiation into committed erythroid progenitors, which mature into erythrocytes [17]. This surge in RBC production helps maintain systemic homeostasis until bone marrow erythropoiesis recovers, highlighting the essential role of EME during infection [18].

Macrophages are central to both erythrophagocytosis and erythropoiesis, making them key players in anaemia development [10,19,20]. A specific subset, erythroblastic island (EBI) macrophages, also known as central macrophages (CMs), forms a niche that supports erythroblast maturation into reticulocytes in both the bone marrow and spleen, especially under stress conditions [21]. These macrophages provide essential growth factors, assist in iron handling, support erythroid differentiation, and clear extruded nuclei from developing red blood cells (RBCs) [22]. During anaemic stress, splenic red pulp macrophages also regulate iron recycling and coordinate emergency erythropoiesis [22]. These processes rely on tightly regulated interactions between progenitors and niche cells, driven by molecular signals that respond to the body’s physiological demands, ensuring an appropriate response to stress [23]. However, EBI macrophages have not been studied in African trypanosomiasis (AT)-associated anemia, which is fueled by a sustained pro-inflammatory response. In this context, IL-10 emerges as a key anti-inflammatory cytokine that helps mitigate the severity of anemia during the course of infection [10,24,25]. Of note, not only during AT, but also during infection with other trypanosomatids, IL-10 has been found to play a regulatory role [26,27]. Yet, the exact contribution of IL-10 to the development of acute anemia associated with AT, particularly its impact on stress erythropoiesis, remains unclear. To address this, we employed genetic and pharmacological approaches to investigate how IL-10 regulates *T. brucei*-associated acute anaemia, focusing on both RBC differentiation and the presence of EBI macrophages in the bone marrow and spleen.

## 2. Materials and Methods

### 2.1. Ethics Statement

All experiments complied with the ECPVA guidelines (CETS n° 123) and were approved by the VUB Ethical Committee for Animal Experiments (Permit Numbers: 14-220-05, 14-220-06, and 20-220-44). All mice were housed in individual ventilated cages, where they had unlimited access to food and water. Mice were monitored daily. Humane endpoints were used during the study, based on weight loss; animals with >25% weight loss were sacrificed using carbon dioxide treatment.

### 2.2. Mouse Strains, Parasites, and Infection

Eight-week-old female wild-type (WT) C57BL/6 mice were purchased from Janvier (Le Genest-Saint-Isle, France). Ubiquitin-GFP (C57BL/6-Tg(UBC-GFP)30Scha/J; stock No: 004353) and *IL10* KO mice (B6.129P2-IL10<tm1Cgn>/J; stock No: 002251), both from the C57BL/6 background, were obtained from Jackson laboratories (Bar Harbor, ME, USA) and bred in the animal facility of the VUB. *T. brucei brucei* AnTat1.1E parasites were a kind gift from N. Van Meirvenne (Institute for Tropical Medicine, Antwerpen, Belgium) and stored at −80 °C as blood aliquots (50 µL) containing 50% Alsever’s solution (Sigma-Aldrich, St. Louis, MO, USA) and 10% glycerol (final *V*/*V*). Then, 950 µL PBS was added, and subsequently, parasites were counted via a hemocytometer and brought to a concentration of 2.5 × 10^4^/mL (stock solution of viable/motile parasites), of which 200 µL was used to infect mice by intraperitoneal (i.p.) injection.

### 2.3. In Vivo Antibody Treatment

Infected 8-week-old female WT C57BL/6 mice were treated with 200 µg of blocking anti-IL-10 receptor (anti-IL-10R, clone 1B1.3A, Bio X Cell) antibody at day 1, 4, and 7 post-infection (p.i.), and/or 300 µg anti-IFN-γ (clone XMG1.2, kind gift from Louis Boon (JJP Biologicals, Warsaw, Poland)) antibody at day 4 and 7 p.i. All injections were performed intraperitoneally (i.p.) in a final volume of 200 µL, and each group consisted of 4–5 mice.

### 2.4. Blood Isolation

By bleeding the mice via tail-cut, 2.5 µL blood was collected and diluted in 500 µL RPMI (RPMI-1640, Gibco, Grand Island, NY, USA) + 5% FCS (fetal calf serum, Gibco). From this, 100 µL was transferred to a FACS-tube, and 10 µL was used to count RBCs and parasitaemia using a hemocytometer. Anaemia was expressed as a percentage of RBCs remaining in infected mice compared to that of non-infected mice or on day 2 p.i.

### 2.5. RBC Clearance Assay

Eight-week-old Ubiquitin-GFP mice were sacrificed, and their blood was isolated with 100 µL heparin (10 times stock 1000 Units/mL, Sigma-Aldrich). From this, 2.5 µL was added to 500 µL RPMI (RPMI-1640, Gibco) + 5% FCS (Gibco) and used to count the RBCs. The remaining blood was washed twice with PBS and centrifuged (10,621× *g*, 7 min, 20 °C). After resuspension of the RBCs with PBS, cells were brought to 1–5 × 10^9^ cells/mL, and the blood (200 µL) was injected intravenously into naive or day 1 post-infection (p.i.) mice. Every second day, RBC numbers were enumerated via hemocytometer, and the remaining cells were analyzed via FACS using a PE-conjugated Ter-119 antibody. Following gating on Ter-119^+^ cells (see Figure S4), two distinct RBC populations could be identified (GFP+ or −). To calculate the RBC clearance, the percentage of GFP^+^ RBCs present at day 1 post-injection was referred to as 100% signal. All other time points were compared with this day.

### 2.6. Preparation of Spleen and BM Suspensions

The spleen and BM (tibia and femur) were isolated and homogenized in 10 mL RPMI + 5% FCS. The cell suspension was filtered using a 40-µm filter, centrifuged (394× *g*, 7 min, 4 °C), and resuspended in 1–5 mL RPMI + 5% FCS. From this cell suspension, 10 µL was taken and added to 190 µL Trypan blue, which was used to count RBCs and white blood cells. Next, the cells were brought to a concentration of 10^7^ cells/mL in RPMI/5% FCS medium. From this stock solution, 10^6^ cells were used for flow cytometric analysis.

### 2.7. Staining and Flow Cytometric Analysis

In total, 100 µL of blood (stock solution: 2.5 µL diluted in 500 µL RPMI + 5% FCS), spleen, or BM (stock solution of 10^7^ cells/mL) was incubated with an Fc-gamma-blocking antibody (clone 2.4G2, kind gift from Louis Boon (JJP Biologicals), 1 µg/10^6^ cells) on ice for 10 min. Then, the cells were stained with an antibody cocktail. Note, when Ubiquitin-GFP C57BL/6 mice were used, the FITC channel was used for detection of the GFP signal. For the blood, two different stainings were used (Table 1: stain 1 and stain 2). For the spleen and BM, four different stainings were used (Table 1: stain 1 (same as for blood), stain 2, stain 3, and stain 4 (similar to stain 3, but in the FITC channel, an isotype control IgG was used). After 20 min incubation in the dark on ice, spleen and BM cells were washed with 1 mL ice-cold RPMI + 5% FCS, centrifuged (394× *g*, 7 min, 4 °C) and resuspended in a final volume of 200 µL RPMI + 5% FCS. The cells were measured on a FACSCanto^TM^ II flow cytometer (BD (Franklin Lakes, NJ, USA) and the results were analyzed with FlowJo_V10 software).

### 2.8. Statistics

Data obtained from flow cytometry was analyzed using FlowJo_V10 and GraphPad Prism 10 software. Statistically significant differences were determined using the Student *t*-test or Two-way ANOVA. Values are expressed as mean ± SEM. Values of *p* ≤ 0.05 are considered statistically significant.

## 3. Results

### 3.1. Blockade of IL-10 Signaling Enhances Acute Anemia Development and Negatively Affects Mature and Immature RBC Formation in the Bone Marrow and Spleen During AT

**Figure 1:**
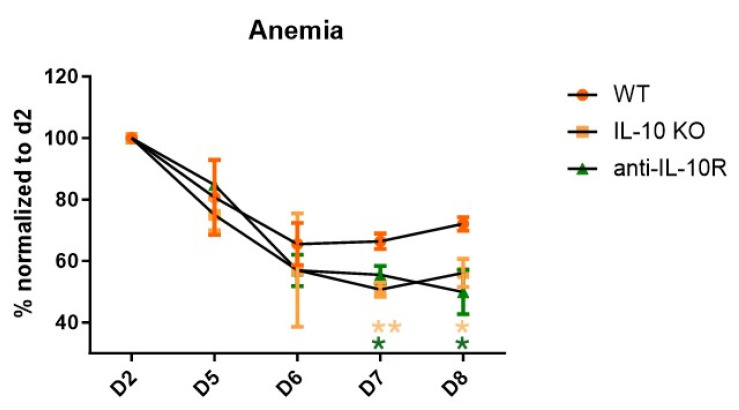
Anaemia development during the acute stage of *T. b. brucei* infection in WT, *IL10*-KO, and anti-IL-10R antibody treated WT mice. Anaemia development during the acute stage of *T. b. brucei* infection in WT and *IL10*-KO as well as anti-IL-10R antibody treated WT mice. Results are representative of two to three independent experiments (*n* = 5) and expressed as SEM. *: *p* < 0.05; **: *p* < 0.01. (* represents WT control versus anti-IL-10R antibody-treated mice; * represents WT control versus *IL10*-KO mice).

By using both IL-10 knockout (*IL10*-KO) mice and anti-IL-10R-blocking antibodies, we assessed the role of IL-10 in acute anaemia during AT and found that disrupting IL-10 signaling consistently exacerbated anaemia compared to the control mice (Figure 1). At day 8 p.i. (i.e., the moment before *IL10*-KO and anti-IL-10R antibody treated mice reach their humane endpoint due to uncontrolled inflammation and severe anaemia), the mice were sacrificed, after which their organs (bone marrow and spleen) were processed into a single-cell suspension and subjected to a flow cytometric analysis to assess the RBC composition (gating strategy Figure S1).

**Figure 2:**
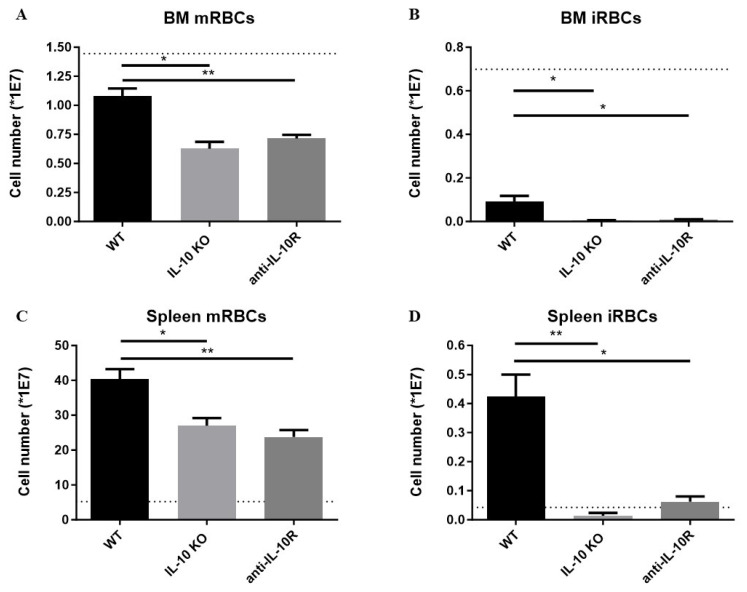
Absolute numbers of mature and immature RBCs in the BM and spleen of *T. b. brucei* infected (day 8 p.i.) WT, anti-IL10R antibody treated WT, and *IL10*-KO mice. Numbers of mature (**A**) and immature (**B**) RBCs in BM (one tibia and femur) at day 8 p.i. Numbers of mature (**C**) and immature (**D**) RBCs in the spleen at day 8 p.i. The dashed lines represent the non-infected mice. Results are representative of two to three independent experiments (*n* = 5) and expressed as SEM. *: *p* < 0.05; **: *p* < 0.01.

At the level of the BM, the numbers of mRBCs and iRBCs declined in all infected groups compared to uninfected mice (dashed line), but infected anti-IL-10R antibody treated and *IL10*-KO mice exhibited significantly reduced numbers of both mRBCs and iRBCs compared to their wild-type (WT) counterparts (Figure 2A,B). Hence, IL-10 deficiency aggravates the impaired erythropoiesis in the BM.

Our previous work on AT revealed that an exaggerated inflammatory response correlates with extramedullary erythropoiesis and with the occurrence of splenomegaly [28]. Hence, we investigated if the increased inflammatory immune response typically observed in infected *IL-10-*KO mice triggered an enhanced extramedullary response [25]. Indeed, infected WT mice exhibit increased splenic numbers of both mRBCs and iRBCs compared to non-infected WT mice (Figure 2C,D). However, although the absence of IL-10/IL-10R signaling is expected to increase the level of inflammation, infected anti-IL-10R antibody treated WT mice and *IL10*-KO mice exhibited significantly reduced numbers of splenic mRBCs and iRBCs compared to their WT counterparts.

Together, these results demonstrate that IL-10 is key in dampening anaemia of inflammation by promoting erythropoiesis within the BM and spleen following African trypanosome infection.

### 3.2. IL-10 Deficiency Negatively Affects the Early Stages of RBC Differentiation Within the Bone Marrow and Spleen During AT

**Figure 3:**
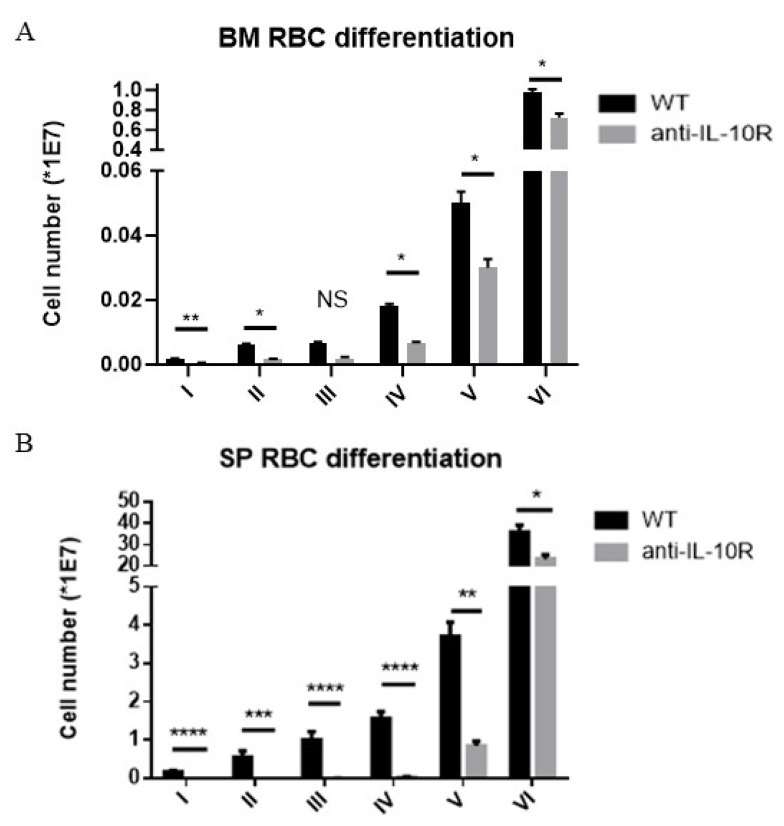
Absolute numbers of the RBC differentiation stages in the bone marrow and spleen of *T. b. brucei* infected (day 8 p.i.) WT and anti-IL-10 receptor antibody treated WT mice. Based on the gating strategy described in Figure S1E, the RBC differentiation stages within the bone marrow (**A**) and spleen (**B**) were identified. Results are representative of two to three independent experiments (*n* = 5) and expressed as SEM *: *p* < 0.05; **: *p* < 0.01; ***: *p* < 0.001; ****: *p* < 0.0001.

In order to acquire insight into which stage of erythropoiesis IL-10 could play a role in, an established gating strategy for erythrocyte maturation stages was used [29] (Figure S1E). It was observed that the early stages (i.e., stage I to IV) of erythropoiesis were impaired at day 8 p.i., in the BM and spleen of anti-IL10R antibody treated infected mice compared to infected WT mice (Figure 3A,B). Similar results were obtained in *IL10*-KO mice (Figure S2). Collectively, these data strongly imply that the absence of IL-10 signaling during the acute phase of the infection adversely affects the early stages of RBC differentiation (stages I to IV) in both the BM and spleen.

### 3.3. Anti-IL-10R Antibody Treated T. b. brucei Infected WT Mice Exhibit Altered Levels of Central Macrophages

Since a subset of macrophages, erythroblastic island (EBI) macrophages, or central macrophages (CMs), is known to play a key role in erythroid development [30], we determined the presence of these cells in the BM and spleen of infected WT mice versus mice with a deficient IL-10/IL-10R signaling. Since anti-IL-10R antibody treated WT mice phenocopied their *IL10*-KO counterparts during infection, all further experiments were performed using the pharmacological approach with the anti-IL-10R blocking antibody.

**Figure 4:**
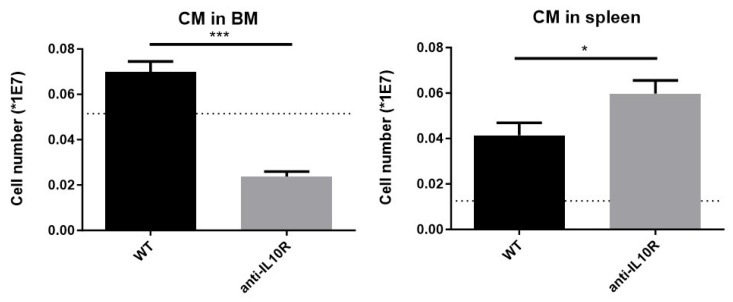
Absolute numbers of central macrophages (CMs) in *T. b. brucei* infected (day 8 p.i.) WT and anti-IL-10R antibody treated WT mice at the level of the BM and spleen. The dashed lines represent the levels in non-infected mice. The gating for CM was as described in Figure S3. Results are representative of two to three independent experiments (*n* = 5) and expressed as SEM. *: *p* < 0.05; ***: *p* < 0.001.

To study CMs, a dedicated gating strategy was used [30], based on their unique F4/80^+^VCAM-1^+^ER-HR3^+^CD169^+^Ly-6G^+^ surface marker profile (Figure S3). At day 8 p.i., the number of CMs in the BM was significantly reduced in infected WT mice treated with an anti-IL-10R antibody compared to infected WT controls, dropping to levels even lower than those observed in non-infected animals (Figure 4, left panel). These data are in line with a reduced presence of early erythrocyte maturation stages in that organ. In contrast, in the spleen, infection led to an increase in the absolute number of CMs in both WT and anti-IL-10R antibody treated WT mice compared to non-infected controls. In addition, there were significantly more CMs in the infected anti-IL-10R antibody treated WT mice as compared to the control group, demonstrating that IL-10 blockade has opposite effects in the BM and spleen (Figure 4, right panel).

### 3.4. Anti-IL-10R Antibody Treated T. b. brucei Infected WT Mice Exhibit an Increased Erythrophagocytosis Capacity and Weight Loss Coinciding with an Increased Myeloid Cell Activation

Besides differences in RBC maturation, an enhanced RBC clearance by myeloid cells may also contribute to the higher anaemia observed in anti-IL-10R antibody treated infected WT mice [19]. To test this, an *in vivo* RBC clearance assay was performed using a previously described method [31]. In brief, GFP^+^ RBCs were isolated from Ubiquitin-GFP transgenic mice, transferred into naïve or day 1 *T. b. brucei*–infected WT mice, and 24 h later, blood was collected to determine the percentage and absolute number of GFP^+^ RBCs (arbitrarily set to 100%) via flow cytometry (gating strategy in Figure S4). Next, the mice were followed from day 4 until day 7 post GFP^+^ RBC injection to monitor both anaemia and the kinetics of GFP^+^ RBC clearance.

**Figure 5:**
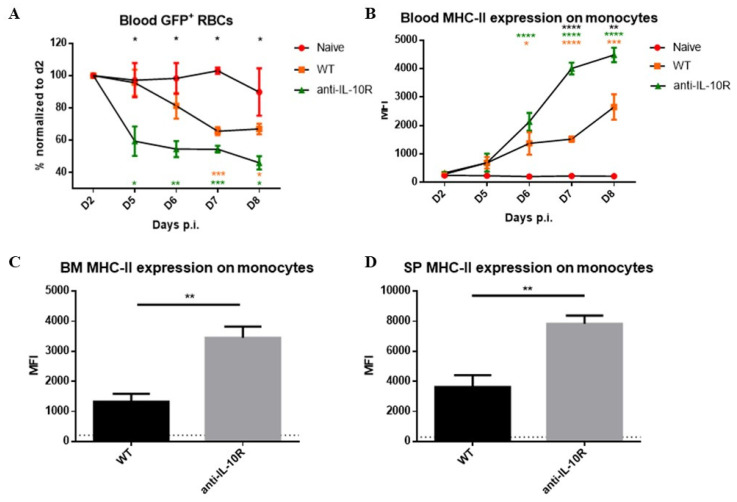
GFP^+^ RBC clearance assay performed in naive and *T. b. brucei-*infected WT and anti-IL-10R antibody treated WT mice, as well as Median fluorescence intensity (MFI) of MHC-II on monocytes at endpoint. (**A**) Kinetics of the GFP signal observed in naïve and *T. b. brucei*-infected control and anti-IL10R antibody treated mice. The gating strategy used to select GFP^+^ RBCs is as described in Figure S4. Values are expressed in SEM of five mice per group and *: *p* < 0.05; **: *p* < 0.01, and ***: *p* < 0.001 (*: WT versus anti-IL-10R antibody-treated mice, *: naive versus WT mice, *: naive versus anti-IL-10R-treated mice). If nothing is mentioned, the differences were not significant. (**B**) Kinetics of the MFI (median fluorescence intensity) of MHC-II on monocytes within the blood during the acute stage of infection in the different groups. MFI of the MHC-II expression on monocytes within the BM (**C**) and spleen (**D**) at day 8 p.i. The gating strategy used to identify monocytes is described in Figure S4. The dashed lines represent levels in the non-infected mice. Results are representative of two to three independent experiments (*n* = 5) and expressed as SEM. *: *p* < 0.05; **: *p* < 0.01; ***: *p* < 0.001; ****: *p* < 0.0001 (For (**A**,**B**): * represent WT versus anti-IL-10R antibody treated mice, * represent naive versus anti-IL-10R treated mice, and * represent naive versus WT mice).

Our data show an enhanced RBC clearance during infection in WT mice compared to non-infected control mice, which became apparent from day 5 p.i. onwards (Figure 5A). Interestingly, the infected anti-IL-10R antibody treated WT mice showed an earlier and enhanced GFP^+^ RBC clearance compared to infected WT animals.

To test if this phenotype is linked to an increased myeloid cell activation that fuels erythrophagocytosis, monocyte MHC-II expression levels were measured as a read-out for their enhanced activation state [32]. Also, the weight loss of the animals was measured as a proxy for increased inflammation. Anti-IL-10R antibody treatment upon infection induced a significantly increased weight loss compared to control infected WT mice as a sign of increased systemic inflammation, which became apparent starting from day 6–7 p.i., corresponding to peak parasitaemia control (Figure S5A). In addition, the monocyte MHC-II expression levels were significantly increased upon infection in WT mice, in blood, BM, and spleen (Figure 5B–D), and this increase was significantly higher in infected anti-IL-10R antibody treated mice. Together, these results strengthen the notion that the anti-IL-10R antibody treatment triggers a stronger activation of myeloid cells, which can, in turn, fuel inflammation and exert an enhanced RBC clearance.

### 3.5. Blocking IFN-γ Attenuates the Effects Observed in T. b. brucei Infected and Anti-IL-10R Antibody Treated WT Mice

IFN-γ is a driving force for the induction of the inflammatory immune response during Trypanosoma infection, resulting in the activation of myeloid cells and the development of acute anaemia [10]. Therefore, to test if the increased IFN-γ levels observed in mice devoid of IL-10 signaling are implicated in the early ablation of extramedullary erythropoiesis, IFN-γ was blocked pharmacologically after priming (around day 3 p.i.). Surprisingly, mice treated with a timely combination of anti-IL-10R and anti-IFN-γ antibodies exhibited similar weight loss as compared to mice receiving anti-IL-10R antibody monotherapy (Figure S5B). In line with this observation, the treatment of infected WT mice with anti-IFN-γ antibodies did not affect the mouse weight loss compared to untreated infected WT mice.

**Figure 6:**
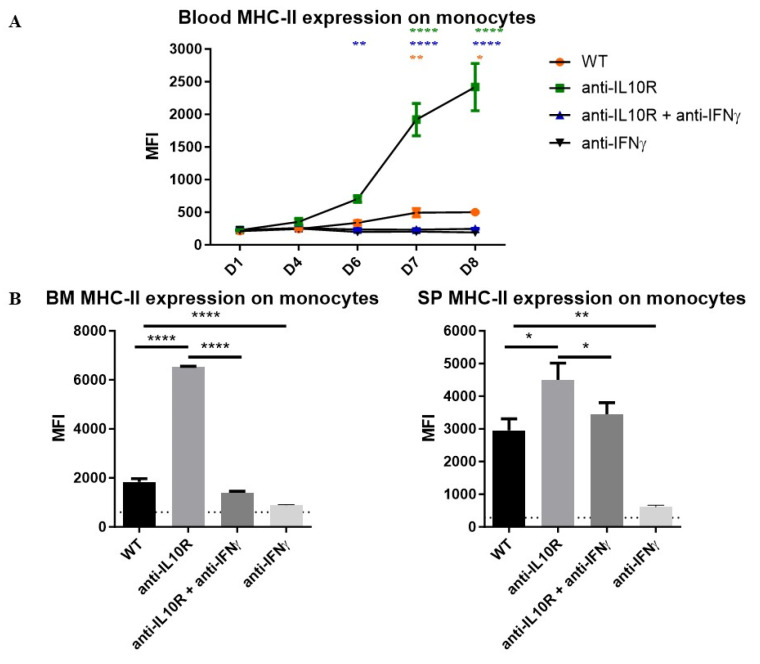
Median fluorescence intensity (MFI) of MHC-II on monocytes from the blood, BM, and spleen in *T. b. brucei*-infected WT, anti-IL-10R antibody treated, anti-IFN-γ antibody treated, and anti-IL-10R + anti-IFN-γ antibody treated WT mice. (**A**) Kinetics of the MFI of MHC-II on monocytes within the blood during the acute stage of infection in the different groups. (**B**) MFI of the MHC-II expression on monocytes within the BM (left panel) and spleen (right panel) at day 8 p.i. The gating strategy used to select for monocytes is as described in Figure S3. The dashed lines represent levels in the non-infected mice. Results are representative of 2–3 independent experiments (*n* = 5) and expressed as SEM. *: *p*< 0.05; **: *p* < 0.01; ****: *p* < 0.0001 (for (**A**): * represent WT versus anti-IL-10R antibody-treated mice, * represent anti-IL-10R + anti-IFN-γ antibody treated versus anti-IL-10R antibody treated mice and * represent anti-IL-10R + anti-IFN-γ antibody treated versus WT mice). The anti-IL-10R + anti-IFN-γ antibody treated and anti-IFN-γ antibody treated mice behaved similarly. If nothing is mentioned, the differences were not significant.

Furthermore, blocking IFN-γ in infected anti-IL-10R antibody treated WT mice completely blocked the upregulation of MHC-II expression on blood monocytes (Figure 6A) and restored MHC-II levels to those seen in untreated WT mice in BM and spleen (Figure 6B). Of note, similar results were obtained with the blockade of IFN-γ in infected WT mice.

**Figure 7:**
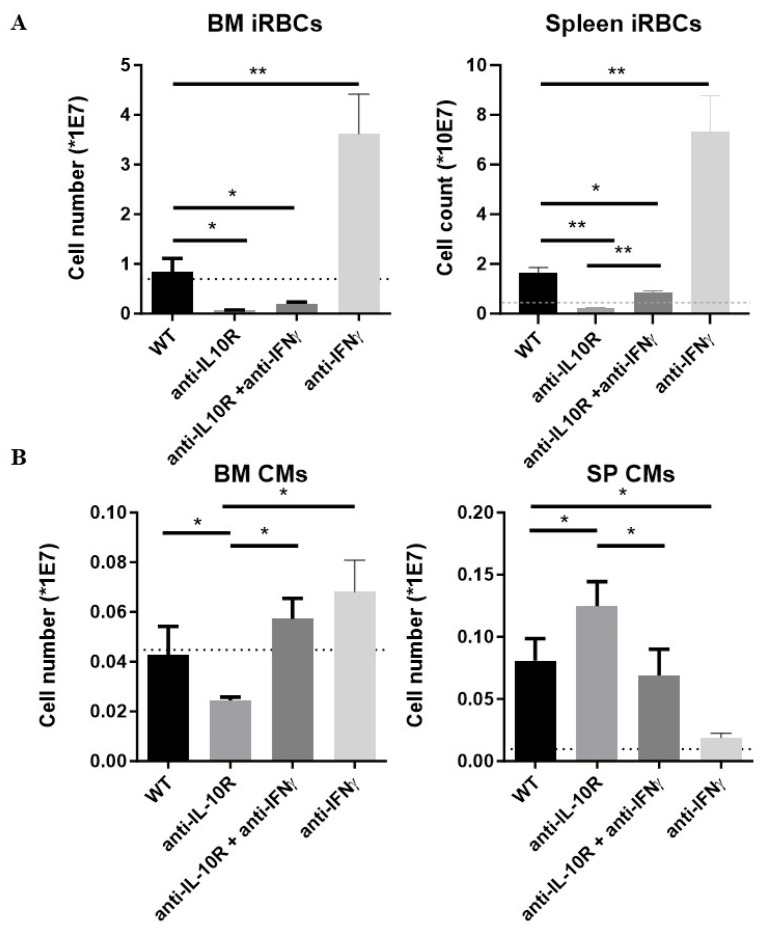
Absolute numbers of immature RBCs and CM within the BM and spleen from *T. b. brucei*-infected WT, anti-IL-10R antibody treated, anti-IFN-γ antibody treated, and anti-IL-10R + anti-IFN-γ antibody treated WT mice. (**A**) Left panel: iRBCs in BM. Right panel: iRBCs in spleen. (**B**) The dashed line represents levels in the non-infected mice. Results are representative of 2–3 independent experiments (*n* = 5) and expressed as SEM. *: *p* < 0.05; **: *p* < 0.01. If nothing is mentioned, the differences were not significant.

In the BM, all groups of infected mice, except the anti-IFN-γ treated WT mice, had reduced numbers of iRBCs compared to non-infected mice, whereby this drop was most pronounced in the anti-IL-10R treated mice and was not restored upon anti-IL-10R and anti-IFN-γ treatment (Figure 7A, left panel). Conversely, in the spleen, the accumulation of iRBCs was partially restored upon anti-IL-10R/anti-IFN-γ combination therapy (Figure 7A, right panel). These mice also exhibited enhanced splenomegaly as compared to mice only treated with an anti-IL-10R antibody; however, they did not reach levels of untreated infected mice (Figure S6). Of note, blocking only IFN-γ in infected WT mice resulted in an expansion of iRBCs both in the BM and spleen (Figure S6).

Finally, CM numbers were investigated. In the BM, the CM numbers were significantly increased in the combination-treated mice as compared to anti-IL-10R antibody treated WT mice, reaching levels similar to the untreated infected WT mice (Figure 7B, left panel). In contrast, splenic CM numbers were significantly reduced in the combination-treated mice as compared to anti-IL-10R antibody treated WT mice, reaching levels similar to the untreated infected WT mice (Figure 7B, right panel). Of note, blocking only IFN-γ in infected WT mice prevented the expansion of infection-induced CM numbers in the spleen and did not significantly affect their numbers in the BM.

Together, these data illustrate the detrimental impact of IFN-γ on weight loss, overactivation of monocytes, spleen iRBCs, as well as the presence of CMs in the BM and spleen.

## 4. Discussion

During the initial phase of *T. b. brucei* infection, a robust pro-inflammatory immune response is initiated to control parasitaemia. Concurrently, this response ultimately promotes T cell-driven IFN-γ production [7], which activates macrophages to secrete pro-inflammatory mediators such as TNF and accelerates red blood cell (RBC) senescence, enhancing their clearance through erythrophagocytosis by myeloid phagocytes. In addition, auto-antibodies directed against phosphatidylserine could also play a role in the induction of anemia to some extent [33]. Hence, anaemia in trypanosome-infected animals is mainly due to self-inflicted damage by a disproportionate immune and/or innate response. To attenuate this strong pro-inflammatory immune response and prevent excessive inflammation-induced pathology [34], and acute anaemia in particular, the host produces IL-10. Since IL-10 has been shown to affect erythropoiesis at several levels [5], and there is an urgent demand for increased erythropoiesis (i.e., stress-induced erythropoiesis), we used different approaches to address its involvement during *T. brucei*-associated acute anaemia development at the level of the BM and spleen.

We showed that IL-10 knockout (*IL10*-KO) mice exhibited more severe anaemia, with a reduction in iRBCs in the BM and the spleen. Detailed analysis of erythroid development showed that the early stages of erythropoiesis (i.e., stages I–IV immature RBCs) were significantly impaired in both the BM and spleen of *IL10*-KO mice. Despite higher levels of systemic inflammation and anaemia, these mice did not show the expected increase in splenic erythropoiesis, which would normally compensate for BM failure. This suggests that IL-10 is essential for supporting early erythroid differentiation and maintaining the function of EME during infection. To confirm that these effects were not due to intrinsic genetic factors in *IL10*-KO mice, WT mice were treated with blocking anti-IL-10 receptor (IL-10R) antibodies. This pharmacological blockade resulted in a similar phenotype, with impaired erythropoiesis in both the BM and spleen, validating that IL-10 signaling is involved in sustaining RBC production during infection. In this context, IL-10 was shown before to act synergistically with EPO to stimulate erythroid differentiation and proliferation in vitro (already at the Burst Forming Unit-Erythroid (BFU-E) and Colony Forming Unit-Erythroid (CFU-E) colony stage) and may be involved in the regulation of normal erythropoiesis in vivo [35]. Hence, IL-10 deficiency may result in an impaired BFU/CFU differentiation during infection.

Given that central macrophages (CMs) are essential to support erythroid maturation, their presence was investigated both at the level of the BM and spleen. During the acute phase of anaemia development, the number of CMs remained constant in infected WT mice, while they were significantly reduced in the BM of anti-IL-10R antibody treated mice. In contrast, although splenic CMs expanded upon infection in both control and anti-IL10R antibody treated mice, their numbers were even higher in the latter group. The fact that in the absence of IL-10 signaling, there are fewer CMs in the BM and more in the spleen compared to control mice might be attributed to the hyperinflammatory state of these mice. Hyperinflammation alters the balance between erythropoiesis/myelopoiesis in favor of myelopoiesis. Indeed, systemic levels of inflammatory cytokines fuel the efflux of monocytic cells into the circulation, and subsequently they accumulate in tissues where they differentiate into tissue-associated macrophages [36]. In this context, using lineage tracing and imaging flow cytometry to analyze the dynamic changes in EBIs during the recovery from anemic stress, it has been shown that the expansion of the splenic niche is due to the recruitment of monocytes into the spleen, which develop into macrophages that form erythroblastic islands [37]. The influx of monocytes into the spleen depends in part on CCR2-dependent signaling and other ligands expressed by spleen resident red pulp macrophages [36,37]. Of note, it remains unclear whether the reduced number of CMs in the bone marrow contributes to the impaired erythropoiesis observed in the anti-IL-10R antibody treated group compared to controls, and whether differences in functional competence exist between the experimental groups at the level of the spleen, as immature RBCs failed to accumulate in the treated group. This raises the possibility that, in the absence of IL-10 signaling, not only are CM numbers affected, but also their ability to support erythroid maturation. However, this requires further investigation.

Given that (i) blocking IL-10 signaling promotes inflammation, (ii) IFN-γ is a key driver of inflammation documented to suppress erythropoiesis (i.e., inhibits erythroid progenitor proliferation and survival) [12], and (iii) the balance between IFN-γ/IL-10 determines the level of AT-associated pathology [10,36,38] and can affect emergency myelopoiesis [39], we investigated if the effects observed upon IL-10 blockade were due to an enhanced IFN-γ production typically observed in these mice. First, we confirmed that anti-IL-10R treated WT mice showed elevated inflammation, evidenced by increased MHC-II expression on monocytes in blood, BM, and spleen. This aligns with IL-10’s known role in downregulating MHC-II and limiting IFN-γ-driven immune activation. Subsequently, to determine whether IFN-γ directly contributes to erythropoietic defects in the absence of IL-10, mice were co-treated with anti-IL-10R and anti-IFN-γ blocking antibodies. This dual blockade normalized monocyte MHC-II expression, indicative of reduced IFN-γ–driven inflammation. The direct effect of IFN-γ on MHC-II expression was confirmed by the fact that the anti-IFN-γ blocking monotherapy also prevented MHC-II upregulation on monocytes. Interestingly, this double blockade also significantly increased splenic immature RBC numbers to levels close to those of WT infected mice and further increased CM numbers, suggesting that IFN-γ plays an important role in EME suppression. However, this double blockade did not increase the number of immature RBCs at the level of the BM, suggesting that IFN-γ is not the sole driver of iRBC expansion. It could, for instance, be that at the level of the BM, the negative effect of IL-10 on the very early stages of RBC differentiation is only partially IFN-γ dependent (affecting only certain stages of erythroid development/differentiation) and that blockade of IFN-γ mainly affects stress-induced splenic erythropoiesis [12]. However, blocking only IFN-γ in infected mice resulted in a massive expansion of immature RBCs both in the spleen and BM, which could be attributed to a direct effect of IFN-γ on different stages of erythropoiesis [12,40,41]. Indeed, IFN-γ is known to tilt the balance between myelopoiesis/erythropoiesis in favor of myelopoiesis, therefore promoting erythropoiesis in its absence. In addition, blocking IFN-γ in infected mice reduced inflammation, as evidenced by the lack of MHC-II upregulation on monocytes, coinciding with alleviated anaemia and reduced splenic CM numbers. Hence, besides increased numbers of splenic iRBCs, there is also less inflammation and thus less suppression on RBC differentiation. Therefore, it is tempting to speculate that the functionality of the CMs is also improved, yet this will require further investigation. Given that it was shown that IFN-γ is a driver of acute anaemia, by promoting erythrophagocytosis, the recovery from acute anaemia seems to rely more on IL-10.

Collectively, these findings underscore the critical role of IL-10 in preserving erythropoiesis during the acute stage of infection by controlling inflammation, particularly IFN-γ, and highlight the complexity of the inflammatory milieu in regulating EME. Without IL-10, uncontrolled inflammation disrupts both BM and splenic erythropoiesis, exacerbating infection-induced anaemia. Our findings suggest, for the first time, that IL-10 signaling and the balance between IL-10 and IFN-γ modulate central macrophages (CMs) in both the BM and spleen during African trypanosome infection, underscoring the need to further explore their functional roles. However, it is important to consider that IL-10 can also induce IFN-γ production and emergency myelopoiesis (EM) [42], hence this IL-10/IFN-γ balance needs to be timely finetuned. Given that CMs support late-stage erythroid maturation, targeting the macrophage compartment may offer a novel therapeutic strategy for anaemia associated with erythropoietic disorders. Finally, elucidating the molecular pathways driving anaemia and hemophagocytosis and/or promoting ‘stress’-erythropoiesis could pave the way for new approaches to disease control and, potentially, ‘acute’ anaemia.

## Figures and Tables

**Table 1:** List of antibodies used with their corresponding clone name and manufacturer.

**Stain 1 (Blood/spleen and bone marrow):**		
**Antibody**	**Clone Name**	**Manufacturer**
CD71 APC	Anti-mouse Clone: R17217	eBioscience (San Diego, CA, USA)
CD71 FITC	Anti-mouse Clone: R17217	eBioscience
TER-119 PE	Anti-mouse Clone: Ter-119	eBioscience
CD45 APC-Cy7	Anti-mouse Clone:30-F11	BioLegend (San Diego, CA, USA)
**Stain 2 (Blood)**		
**Antibody**	**Clone Name**	**Manufacturer**
TER-119 PE	Anti-mouse Clone: Ter-119	eBioscience
Ly6C APC	Anti-mouse clone: ER-MP20	BioRad (Hercules, CA, USA)
CD45 APC-Cy7	Anti-mouse Clone:30-F11	BioLegend
CD11b PE-Cy7	Anti-mouse/human Clone: M1/70	BioLegend
Ly6G PerCP-Cy5.5	Anti-mouse Clone: 1A8	Tonbo Biosciences (San Diego, CA, USA)
B220 BV510	Anti-mouse/human Clone: RA3-6B2	BioLegend
MHC-II BV421	Anti-mouse Clone: M5/114.15.2	BioLegend
**Stain 2 (Spleen and bone marrow)**		
**Antibody**	**Clone Name**	**Manufacturer**
TER-119 PE	Anti-mouse Clone: Ter-119	eBioscience
CD44 APC	Anti-human/mouse Clone: IM7	eBioscience
CD45 APC-Cy	Anti-mouse Clone:30-F11	BioLegend
CD11b PE-Cy7	Anti-mouse/human Clone: M1/70	BioLegend
MHC-II PerCP-Cy5.5	Anti-mouse Clone: M5/114.15.2	BioLegend
B220 BV510	Anti-mouse/human Clone: RA3-6B2	BioLegend
Ly6C BV421	Anti-mouse Clone: HK1.4	BioLegend
**Stain 3 (Spleen and bone marrow)**		
**Antibody**	**Clone Name**	**Manufacturer**
ER-HR3 FITC	Anti-mouse (Alexa) clone: ER-HR3	BioRad
F4/80 PE	Anti-mouse clone CL:A3-1	BioRad
Ly6C APC	Anti-mouse clone: ER-MP20	BioRad
CD45 APC-Cy	Anti-mouse Clone:30-F11	BioLegend
CD11b PE-Cy7	Anti-mouse/human Clone: M1/70	BioLegend
Ly6G PerCP-Cy5.5	Anti-mouse Clone: 1A8	Tonbo Biosciences
B220 BV510	Anti-mouse/human Clone: RA3-6B2	BioLegend
MHC-II BV421	Anti-mouse Clone: M5/114.15.2	BioLegend
**Stain 4 (Spleen and bone marrow)**		
**Antibody**	**Clone Name**	**Manufacturer**
Isotype control FITC	Anti-mouse IgG1 Clone: eBRG1	Serotec (Kidlington, UK)
F4/80 PE	Anti-mouse clone CL:A3-1	BioRad
Ly6C APC	Anti-mouse clone: ER-MP20	BioRad
CD45 APC-Cy	Anti-mouse Clone:30-F11	BioLegend
CD11b PE-Cy7	Anti-mouse/human Clone: M1/70	BioLegend
Ly6G PerCP-Cy5.5	Anti-mouse Clone: 1A8	Tonbo Biosciences
B220 BV510	Anti-mouse/human Clone: RA3-6B2	BioLegend
MHC-II BV421	Anti-mouse Clone: M5/114.15.2	BioLegend

## Data Availability

All material is available to interested researchers upon request.

## Supplementary Materials

The following supporting information can be downloaded at: https://www.mdpi.com/article/10.3390/pathogens14121276/s1.

## Abbreviations

The following abbreviations are used in this manuscript:

AT	African trypanosomosis
AAT	Animal African Trypanosomosis
BFU-E	Burst Forming Unit-Erythroid
CFU-E	Colony Forming Unit-Erythroid
CM	Central macrophage
EBI	Erythroblastic island
EM	Emergency myelopoiesis
EME	Extramedullary erythropoiesis
EPO	Erythropoietin
FCS	Fetal calf serum
GFP	Green Fluorescent protein
HAT	Human African trypanosomosis
iRBCs	Immature RBCs
mRBC	Mature RBCs
RBCs	Red blood cells
SEPs	Stress erythroid progenitors
