# Supplementary material for: IL-10 Plays a Critical Role in Mitigating Acute Anaemia Development During African Trypanosome Infection
Source: Pathogens. 2025 Dec 12;14(12):1276. doi: 10.3390/pathogens14121276 (PMC12735828; doi:10.3390/pathogens14121276)
Supplement: Supplementary file 1 [file pathogens-14-01276-s001.zip › pathogens-3931119-supplementary.pdf]

## Supplemental data:

## Figure S1:

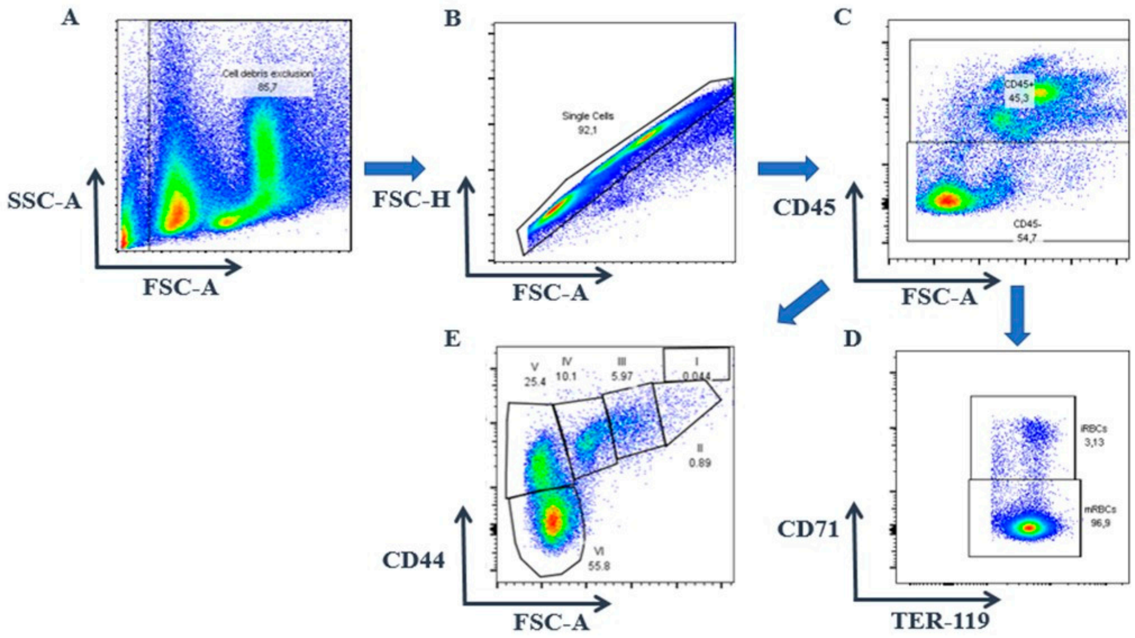


**Figure S1: Representative gating strategy used to study RBC differentiation in WT and IL-10 "inactivated" mice at the level of the BM and spleen.** A) The cell debris was excluded by making a gate in the graph SSC-A in function of FSC-A. B and C) After selecting single cells, CD45 was used to distinguish between immune cells ($CD45^{high}$) and RBCs ($CD45^{low}$). D) Then, within the $CD45^{low/-}$ population, a CD71 versus TER-119 plot was made whereby TER-119 (*i.e.,* glycophorin A-associated protein) is highly specific for erythroid cells (Kina et al., 2000) and CD71 (*i.e.,* transferrin receptor) mediates the uptake of transferrin-iron complexes. The highest level of expression of CD71 is observed on reticulocytes (Marsee et al., 2010). Hence, two main RBC populations could be discriminated: mature RBCs ($TER\text{-}119^{+}CD71^{-}$) and immature RBCs ($TER\text{-}119^{+}CD71^{+}$). E) Alternatively, within the $CD45^{low/-}$ population, CD44 was plotted in function of FSC-A (Liu et al., 2013), which allows to discriminate different stages of erythroid development, starting from nucleated erythroblasts (I (pro), II (Basophilic), III (polychromatic) and IV (orthochromatic)) till enucleated erythrocytes (V (reticulocyte) and VI (erythrocyte)). From stage I to IV, the cell size decreases progressively and stage IV will expel their nuclei in order to produce stage V. The last stage, stage VI, corresponds to mature RBCs, which are at this point ready to enter the blood stream.

**Figure S2:**

A

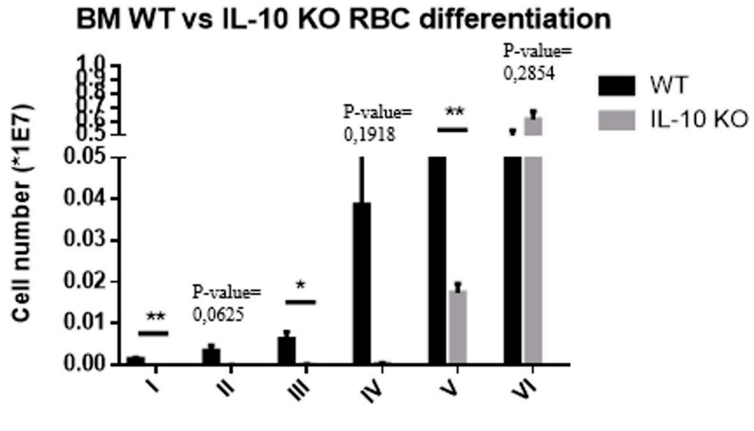


B

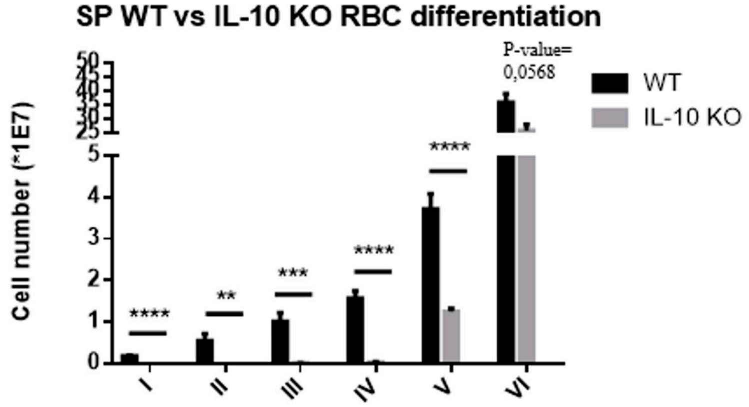


**Figure S2:** **Bar charts showing the absolute numbers of the RBC differentiation stages in BM and spleen of *T. b. brucei* infected (day 8 p.i.) WT and IL-10-*KO* mice.** The gating strategy described in figure 1E was used to identify the different RBC stages within the bone marrow (A) and spleen (B). Results are representative of 2–3 independent experiments (n = 5) and expressed as SEM. *: p < 0.05; **: p < 0.01; ***: p < 0.001; ****: p < 0.0001. If nothing is mentioned, the differences were not significant.

**Figure S3:**

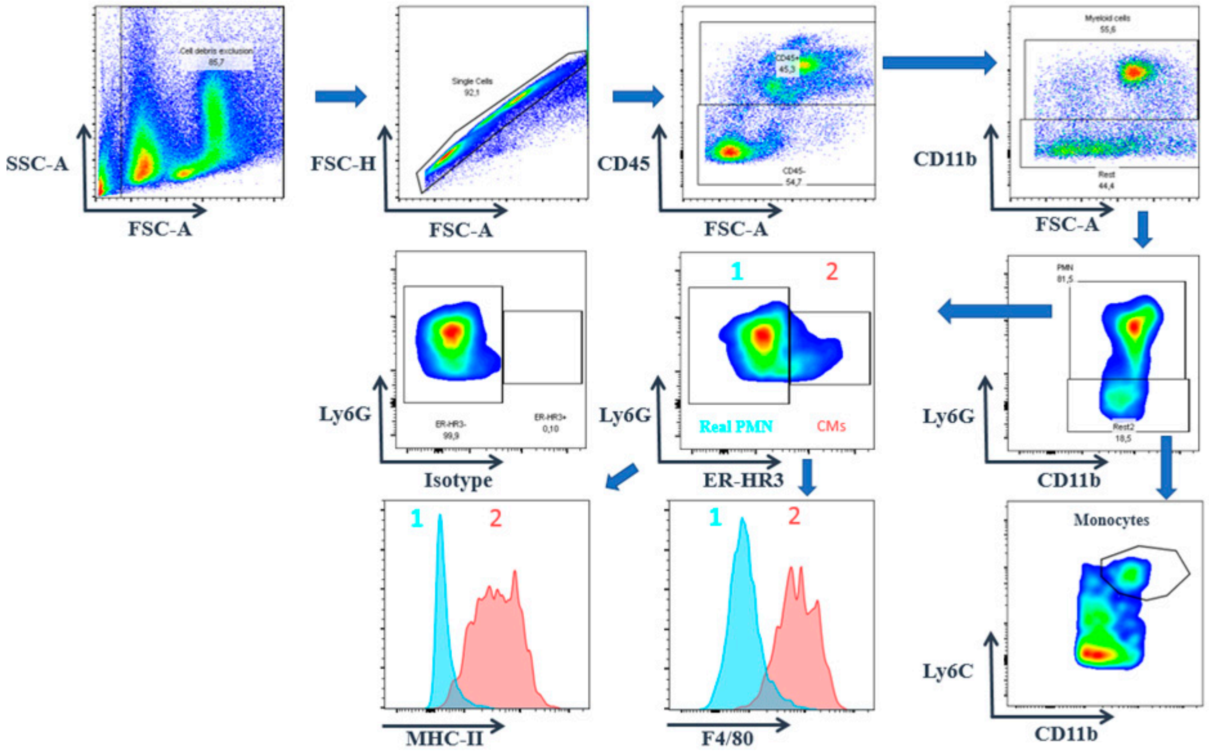


**Figure S3: Representative bone marrow gating strategy used to select central macrophages (CMs)**. First, the cell debris and doublets were excluded. Then, CD45$^{High}$ cells were selected and subsequently, CD11b$^{+}$ cells were gated from a CD11b versus FSC-A plot. Next, Ly6G was plotted in function of CD11b, whereby it was possible to distinguish polymorphonuclear (PMN, granulocytes) cells that are Ly6G$^{+}$CD11b$^{+}$ from the rest. Subsequently, the PMN population was plotted in a Ly6G versus ER-HR3 or Ly6G versus isotype graph. Hereby, two populations could be discriminated, whereby in contrast to the ER-HR3$^{-}$ population (i.e. real PMN, 1) the ER-HR3$^{+}$ population (2) was also found positive for F4/80 and MHC-II expression, indicating that these are more macrophage-like cells (*i.e.*, CMs). A similar gating strategy can be used for the spleen.

**Figure S4:**

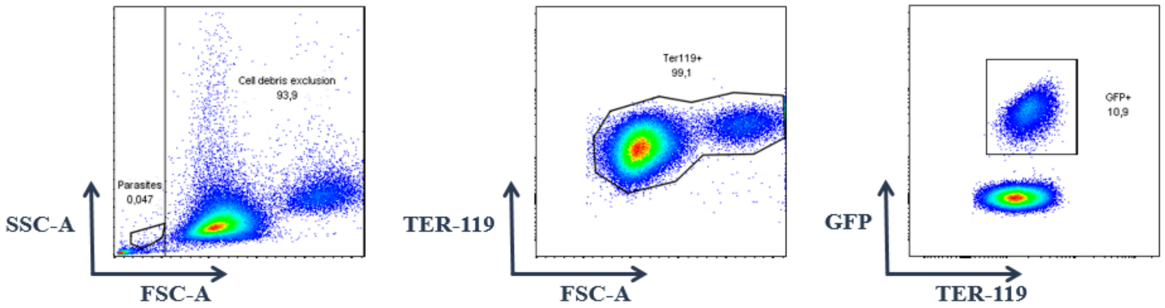


**Figure S4: Representative gating strategy used to select GFP$^{+}$ RBCs within the blood.** $10^{9}$ GFP$^{+}$ RBCs from Ubiquitin-GFP mice were injected i.v. into naïve and *T. b. brucei* infected (day 1 p.i.) WT and anti-IL-10R antibody treated mice. The next day, blood was collected and analyzed via flow cytometry. First, cell debris was excluded in an SSC-A versus FSC-A plot. Then, in a Ter-119 versus FSC-A plot a gate was made to select on TER-119$^{+}$ cells (*i.e.,* RBCs). Finally, from a GFP versus TER-119 graph, the GFP$^{+}$ cells were selected.

## Figure S5:

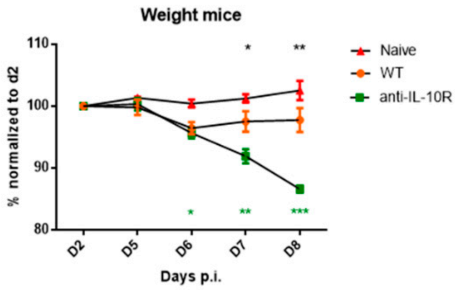


(**A**)

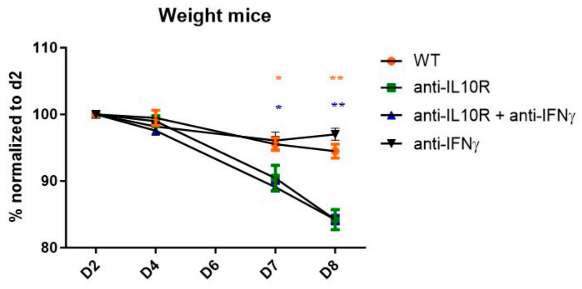


(**B**)

**Figure S5: Weight losses in naive and *T. b. brucei* infected WT, anti-IL-10R antibody as well as anti-IFN-γ antibody and anti-IL-10R + anti-IFN-γ antibody treated mice.** Weight losses in (A) naive and *T. b. brucei* infected WT and anti-IL-10R antibody treated mice, and (B) naive and *T. b. brucei* infected WT and anti-IL-10R + anti-IFN-γ antibody treated mice. Results are representative of 2–3 independent experiments (n = 5) and expressed as SEM. *: $p < 0.05$; ***: $p < 0.001$; ****: $p < 0.0001$ (*: WT versus anti-IL-10R antibody treated mice, *: naive versus anti-IL-10R antibody treated mice, *: anti-IL-10R + anti-IFN-γ antibody treated versus anti-IFN-γ antibody treated mice, *: WT versus anti-IL-10R antibody treated mice). If nothing is mentioned, the differences were not significant.

Figure S6:

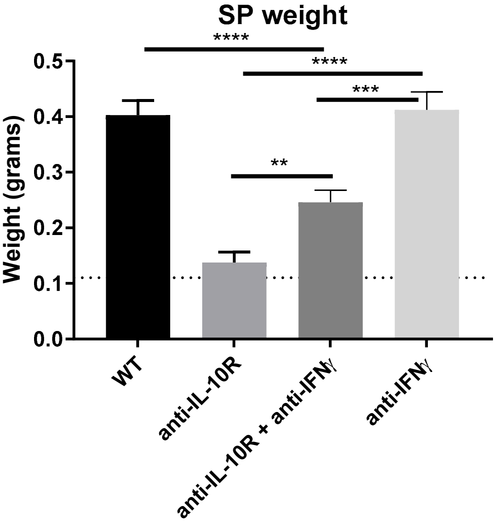


**Figure S6: Spleen weight of naive and *T. b. brucei* infected WT, anti-IL-10R antibody, anti-IFN-γ antibody, as well as anti-IL-10R + anti-IFN-γ antibody treated mice at day 8 post *T. b. brucei* infection.** The dashed lines represent levels in the non-infected mice. Results are representative of 2–3 independent experiments (n = 4-5) and expressed as SEM. **: $p < 0.01$; ***: $p < 0.001$, ****: $p < 0.0005$.
